# Identification of Novel microRNA Profiles Dysregulated in Plasma and Tissue of Abdominal Aortic Aneurysm Patients

**DOI:** 10.3390/ijms21134600

**Published:** 2020-06-28

**Authors:** Emma Plana, Laura Gálvez, Pilar Medina, Silvia Navarro, Victoria Fornés-Ferrer, Joaquín Panadero, Manuel Miralles

**Affiliations:** 1 Angiology and Vascular Surgery Service, La Fe University and Polytechnic Hospital, 46026 Valencia, Spain; lauragn_124@hotmail.com (L.G.); miralles_manher@gva.es (M.M.); 2 Haemostasis, Thrombosis, Arteriosclerosis and Vascular Biology Research Group, Medical Research Institute Hospital La Fe (IIS La Fe), 46026 Valencia, Spain; medina_pil@gva.es (P.M.); navarro_silros@gva.es (S.N.); 3 Data Science, Biostatistics Unit, Medical Research Institute Hospital La Fe (IIS La Fe), 46026 Valencia, Spain; victoriafornes@hotmail.com; 4 Genomic-Bioinformatic Unit, Medical Research Institute Hospital La Fe (IIS La Fe), 46026 Valencia, Spain; btcpar@gmail.com; 5 Department of Surgery, University of Valencia, 46010 Valencia, Spain

**Keywords:** abdominal aortic aneurysm, microRNAs, miRNAs, biomarkers, Thrombospondin-2, vascular disease

## Abstract

microRNAs (miRNAs) are small RNAs that regulate different biological processes. Our objective was to identify miRNAs dysregulated in plasma and tissue of patients with abdominal aortic aneurysm (AAA) and explore new potential targets involved in AAA. Fifty-seven subjects were recruited for a plasma study (30 AAA patients, 16 healthy volunteers and 11 patients with atherosclerosis). The expression level of 179 miRNAs was screened in plasma from a subset of samples, and dysregulated miRNAs were validated in the entire study population. Dysregulated miRNAs were also quantified in aortic tissue of 21 AAA patients and 8 organ donors. Applying a gene set enrichment analysis, an interaction map of dysregulated miRNAs and their targets was built, and selected targets were quantified in tissue samples. miR-27b-3p and miR-221-3p were overexpressed in plasma of AAA patients compared with healthy controls, 1.6 times and 1.9 times, respectively. In AAA tissue, six miRNAs (miR-1, miR-27b-3p, miR-29b-3p, miR-133a-3p, miR-133b, and miR-195-5p) were underexpressed from 1.6 to 4.8 times and four miRNAs (miR-146a-5p, miR-21-5p, miR-144-3p, and miR-103a-3p) were overexpressed from 1.3 to 7.2 times. Thrombospondin-2, a target of miR-195-5p, was increased in AAA tissue and negatively correlated with the expression of miR-195-5p, suggesting their involvement in a common regulatory mechanism.

## 1. Introduction

Abdominal aortic aneurysm (AAA) is a permanent arterial dilatation of over 50% of the normal diameter. The prevalence in the European population ranges from 4.1% to 11.5% [1]. Smoking is one of the main risk factors, together with male sex, age, hypertension (HTN), chronic obstructive pulmonary disease, hyperlipidemia, and family history of the disorder [2]. Several molecular mechanisms, such as proteolytic degradation of aortic wall connective tissue, inflammation, immune response, or biomechanical deterioration of aortic tissue, are involved in AAA pathogenesis [3]. However, those mechanisms are not completely understood and ought to be further studied. In consequence, efforts are being made to elucidate the role of new molecules, such as non-coding RNAs, in AAA formation and progression.

microRNAs (miRNAs) are small (19–24 nucleotides) and highly conserved non-coding RNAs involved in gene regulation by binding to the 3′ untranslated region of a target mRNA, thus restraining its translation. miRNAs are involved in several processes, such as cellular differentiation, apoptosis, or tumorigenesis [4]. In addition, miRNA have been identified as regulators of several cardiovascular diseases [5]. Moreover, miRNAs are stable and present in biofluids, such as plasma, serum, saliva, or milk, and have many features of ideal biomarkers [6]. Thus, research efforts are being focused on the use of these molecules in the context of different pathologies.

Most of the studies that investigate the role of miRNAs in AAA have been performed in vitro or in animal models and have evidenced the importance of these molecules in AAA physiopathology [7]. Regarding research of miRNAs in human AAA, works are focused on the detection of miRNA expression, either in tissue or in biofluids, but limited studies compare the expression pattern in both tissue and biofluids [8,9,10,11,12]. Moreover, results are often not reproducible among publications, probably due to the lack of well-established protocols and the variability of controls used [13]. Therefore, additional studies about miRNAs in human AAA are needed to elucidate their role in the pathology and their use as biomarkers and/or therapeutic targets. With this purpose, we aimed to identify a profile of dysregulated miRNAs in plasma of AAA patients to explore their role as potential AAA biomarkers. Moreover, we evaluated the expression status of these miRNAs, their network interactions and their potential targets in an independent group of surgical AAA specimens with the aim to discover new molecules that could be involved in the molecular regulatory mechanisms of AAA.

## 2. Results

### 2.1. Screening Stage

Table 1 shows the characteristics of the patients (AAA) (*n* = 7) and healthy volunteers (CTL) (*n* = 7) included in the screening stage. These samples were representative of the whole sample set in terms of risk factors and age (data not shown). All participants in the screening stage were male, and there were no significant differences in diabetes mellitus (DM), dyslipidemia (DL), hypertension (HTN), or smoking habits between CTL and AAA. Healthy volunteers were younger than AAA patients (62 ± 3 vs. 72 ± 7 years, *p* < 0.05).

In this sample set, we screened the expression level of 179 miRNAs using a predesigned plasma panel, which included 5 stable miRNAs tested as potential endogenous normalizers and 5 internal quality controls (spike-ins). Potential normalizers were miR-423-5p, miR-425-5p, miR-93-5p, miR-103a-3p, and miR-191-5p. According to the RefFinder comprehensive tool [14], miR-191-5p was rendered as the most stable miRNA in plasma samples. miRNAs that showed expression values of cycle threshold (CTs) above 36 in most of the samples were discarded (*n* = 39). The expression level of the remaining 139 miRNAs was normalized with the expression level of miR-191-5p.

**Figure 1:**
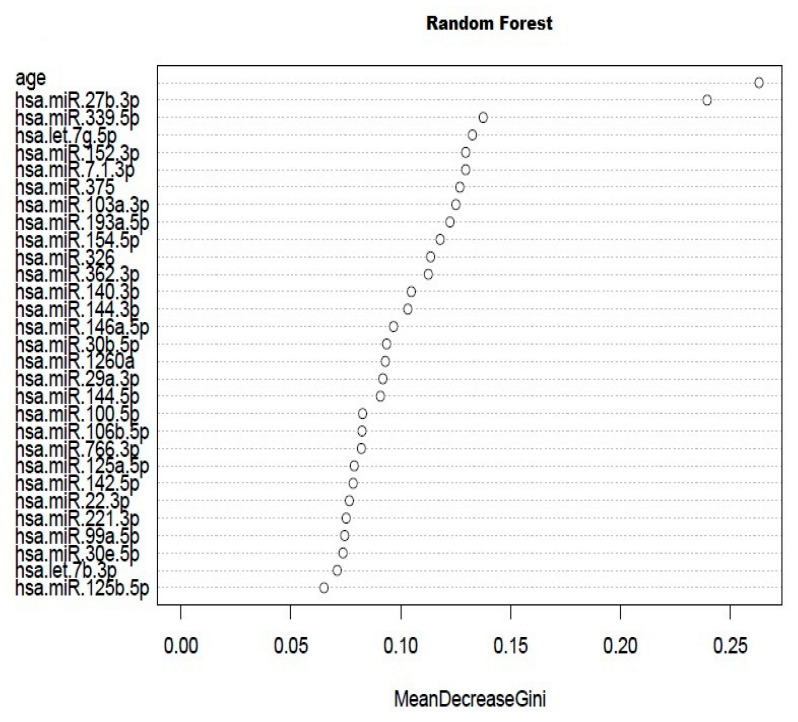
Random Forest graph from microRNAs (miRNAs) quantified in plasma in the screening stage. The Random Forest was performed with 144 variables, including 139 miRNAs, age, hypertension (HTN), diabetes mellitus (DM), dyslipidemia (DL), and smoking habits.

A Random Forest was performed with 144 variables, including 139 miRNAs, age, HTN, DM, DL, and smoking habits [AUC (area under curve) = 0.55, Figure S1]. This algorithm ranked all the variables according to their importance. The first 30 variables listed by Random Forest are shown in Figure 1 (Table 2). According to this classification, age and miR-27b-3p were the most relevant variables to discriminate AAA and CTL. Thus, miRNA-27b-3p was selected for its quantification in the whole plasma sample set (*n* = 57). Due to the multifactorial nature of AAA, the identification of a single specific biomarker has been, to date, unsuccessful, and a combination of several markers may be more reliable. With this purpose, the remaining listed miRNAs were deeply studied. Those miRNAs with a fold-change >2 or <−2, were selected for further study. From them, miR-326, miR-193a-5p, and let-7b-3p were discarded since levels of expression were over the threshold in most samples (CT > 35). Among the rest of the miRNAs, an exhaustive bibliographic search was carried out regarding their reported relation to aneurysm, vascular diseases, or vascular remodeling; as a consequence, three miRNAs (miR-103a-3p, miR-146a-5p, and miR-221-3p) were also studied [7,9,15,16,17,18,19]. Finally, miR-195-5p was also quantified because of its relevance in the literature [20].

### 2.2. Quantification of Selected miRNA in Plasma Samples

The characteristics of the 57 participants included in the complete plasma study are shown in Table 1. Differences were found in age (*p* < 0.001), DM (*p* = 0.046), and HTN (*p* = 0.035) between AAA and CTL. Additionally, differences were found in age (*p* = 0.038), DM (*p* = 0.028), and smoking habits (*p* = 0.019) between carotid endarterectomy (CE) and CTL and also between CE and AAA in age (*p* = 0.019) and HTN (*p* = 0.047). Gender was not explored as a cardiovascular risk factor because all patients included were men. Regarding medication, significant differences were found in the use of statins between AAA and CTL (*p* = 0.004) and between CTL and CE (*p* < 0.001). In addition, the use of acetyl salicylic acid (ASA) was more frequent in CE than in CTL (*p* = 0.006). Two miRNAs, miR-7-1-3p and miR-195-5p, were undetectable (CT > 35) in a high number of plasma samples and were, therefore, discarded. Seven miRNAs were overexpressed in AAA plasma with fold-changes ranging between 1.49 and 2 (Table 3).

**Figure 2:**
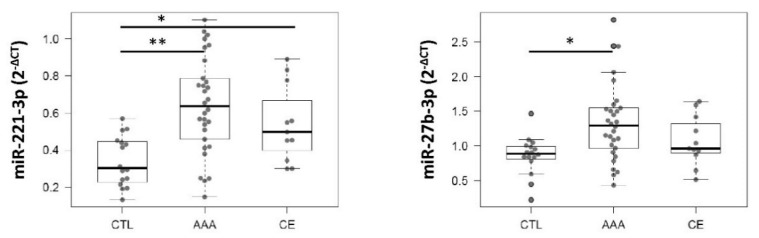
Boxplots of the expression levels of miR-221-3p and miR-27b-3p in plasma samples of abdominal aortic aneurysm (AAA) patients compared with healthy controls and carotid endarterectomy (CE) group. Significant differences are shown as *, *p* < 0.05 and **, *p* < 0.001. CTL, control group (*n* = 16); AAA, aneurysm patients (*n* = 30); CE, patients with severe atherosclerosis (*n* = 11).

In the plasma study, the expression level of each miRNA was compared among the different groups studied (AAA, CTL, and CE) with a multivariable linear regression model, including cardiovascular risk factors (age, HTN, DL, DM, and smoking) as confounding variables. After adjusting, miR-221-3p (*p* < 0.001; 95% Confidence Interval (CI), [0.145, 0.521]) and miR-27b-3p (*p* = 0.047; 95%CI, [0.014, 0.841]) remained significantly overexpressed in AAA plasma compared with CTL. No significant differences in the expression levels of these miRNAs were observed between AAA and CE patients (Figure 2).

### 2.3. Quantification of Selected miRNA in Tissue Samples

**Figure 3:**
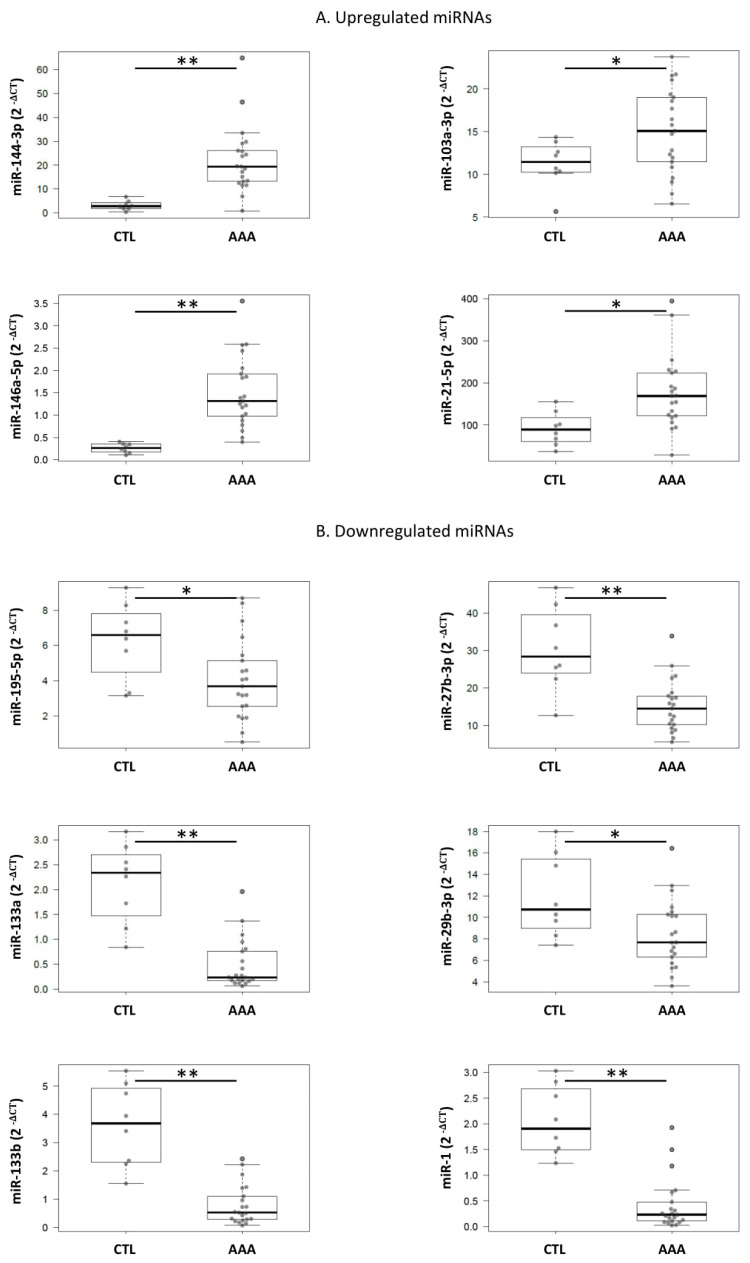
Boxplots of the expression levels of the 10 dysregulated miRNAs in AAA patients compared with organ donors tissue samples. (**A**) miRNAs upregulated in AAA tissue samples (miR-146a-5p, miR-21-5p, miR-144-3p, and miR-103a-3p); (**B**) miRNA downregulated in AAA tissue samples (miR-27b-3p, miR-1, miR-29b-3p, miR-133a-3p, miR-133b, and miR-195-5p). CTL, control group (*n* = 8); AAA, abdominal aortic aneurysm patients (*n* = 21). Values were adjusted with a linear regression model. Significant differences are shown as *, *p* < 0.05 and **, *p* < 0.001.

The aforementioned 11 miRNAs (miR-27b-3p, miR-152-3p, miR-7-1-3p, miR-375, miR-103a-3p, miR-144-3p, miR-146a-5p, miR-1260a, miR-221-3p, miR-99a-5p, and miR-195-5p) together with miR-1, miR-133a, miR-133b, miR-21-5p, miR29b-3p, and miR-155-5p, because of their previously reported relevance [9,15,21,22,23,24], were quantified in an independent set of 29 aortic tissue samples from a different group of AAA patients and organ donors. Clinical characteristics of AAA patients included in the tissue study are shown in Table 1. The expression level of the tissue miRNAs was normalized with the expression level of miR-423-5p being the most stably expressed miRNA in tissue according to RefFinder. The expression of miR-144-3p, miR-146a-5p, miR-21-5p, and miR-103a-3p was higher in AAA tissue than in healthy tissues 7.2-fold (*p* < 0.001; 95%CI, [8.656, 29.348]), 5.8 (*p* < 0.001; 95%CI, [0.662, 1.84]), 1.9 (*p* = 0.012; 95%CI, [20.360, 151.689]), and 1.3-fold (*p* = 0.05, 95%CI, [0.007, 7.705]), respectively. Additionally, miR-1, miR-133b, miR-133a-3p, miR-27b-3p, miR-195-5p, and miR-29b-3p were downregulated in AAA tissue 4.8-fold (*p* < 0.001; 95%CI, [−2.105, −1.150]); 4.6 (*p* < 0.001; 95%CI, [−3.637, −2.002]); 4.4 (*p* < 0.001; 95%CI, [−2.150, −1.135]); 2 (*p* < 0.001; 95%CI, [−22.319, −8.149]); 1.6 (*p* = 0.023; 95%CI, [−4.163, −0.333]), and 1.4-fold (*p* = 0.018; 95%CI, [−6.373, −0.645]) with respect to healthy tissue (Figure 3, Table 4). miR-1260, miR-375, and miR-7-1-3p were discarded because of a poor expression level in tissue.

### 2.4. Identification of miRNA Targets

**Figure 4:**
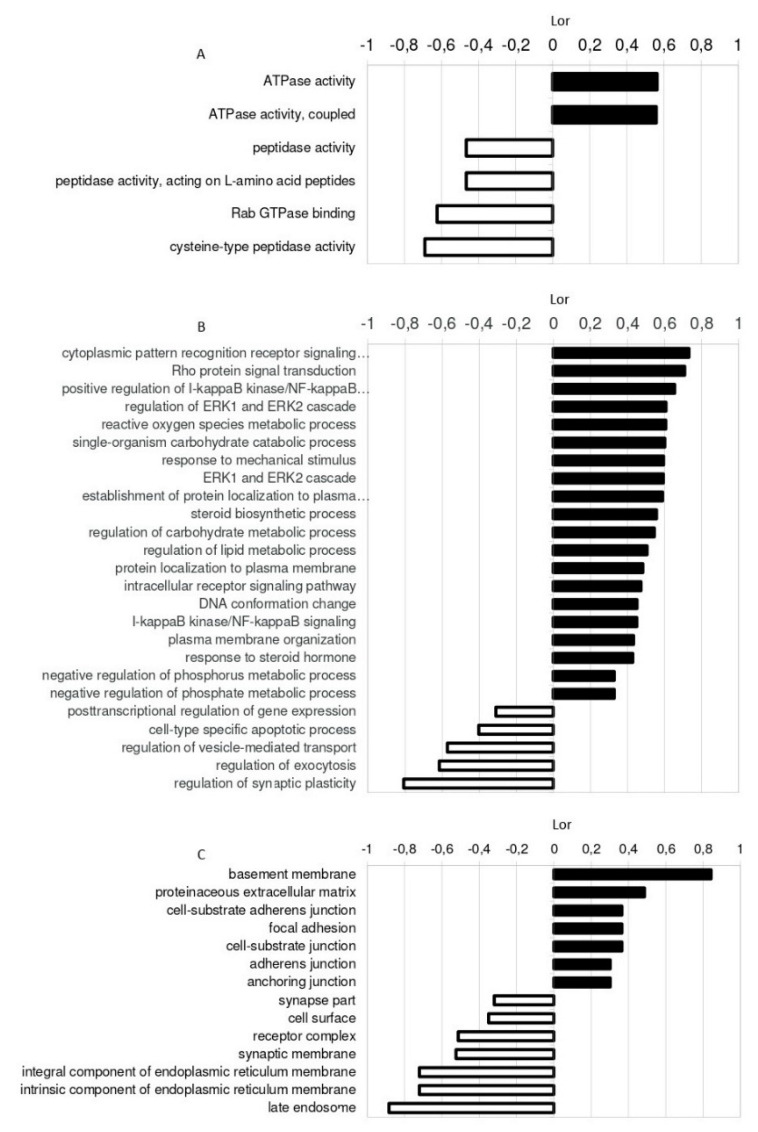
Gene Ontology (GO) enrichment analysis of nine miRNAs (miR-144-3p, miR-146a-5p, miR-21-5p, and miR-103a, miR-1, miR-133b, miR-27b-3p, miR-195-5p, and miR-29b-3p) dysregulated in tissue of AAA patients compared with CTL. miR-133a was not included in the analysis because its high homology with miR-133b causes the sharing of most of the targets. Significant enriched (**A**) molecular functions (MF), (**B**) biological processes (BP), and (**C**) cellular components (CC) are represented. Bars represent the degree of regulation measured by the logarithm of ratio (Lor).

A gene set enrichment analysis (GSEA) of the dysregulated miRNAs in AAA tissue was performed. Briefly, target genes were obtained using a computational analysis that compiles the information allocated in the TargetScan, miRDB, and miRTarBase databases. This search renders two types of results, validated targets (when previous studies have experimentally validated this regulation) and predicted targets (those being theoretically estimated based on the free binding energy between a miRNA and a putative target mRNA sequence). The functional profile of tissue dysregulated miRNAs was characterized using the Gene Ontology (GO) database [25,26] according to their predicted and validated targets. The main molecular functions (MF), biological processes (BP), and cellular components (CC) regulated are shown in Figure 4.

**Figure 5:**
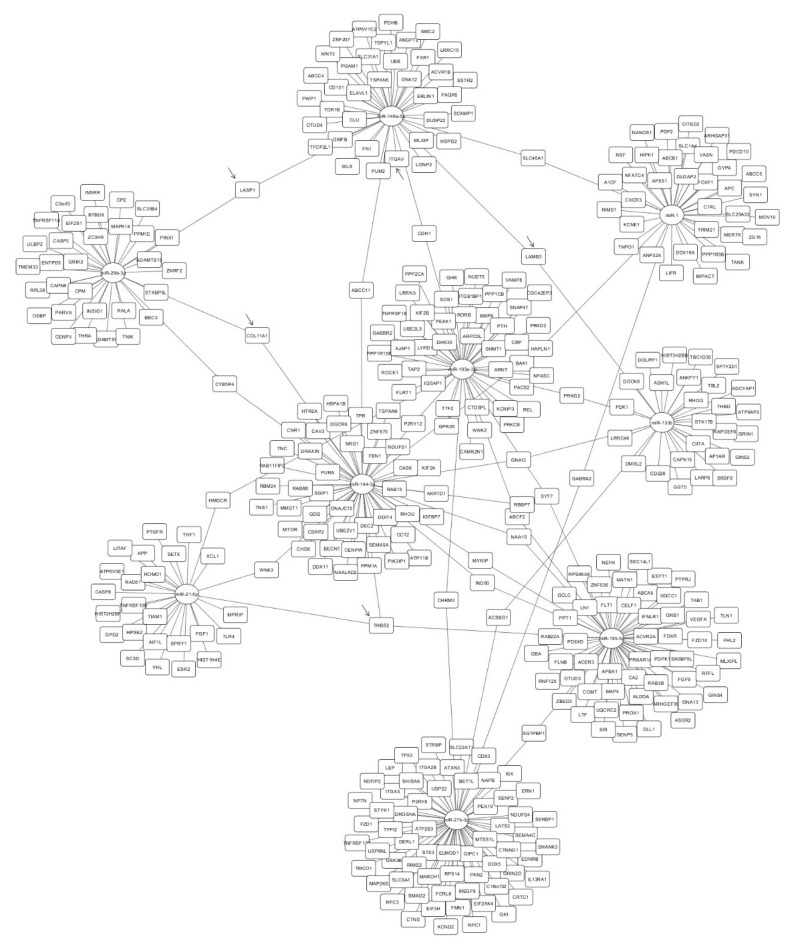
Interaction network of the nine miRNAs (miR-144-3p, miR-146a-5p, miR-21-5p, and miR-103a, miR-1, miR-133b, miR-27b-3p, miR-195-5p, and miR-29b-3p) dysregulated in AAA tissue and their target genes after applying the GSEA. miR-133a was not included because, due to its high homology with miR-133b, they share most of the targets. miRNAs are represented in circles and targets in squares. Selected targets to be quantified in tissue samples are marked with an arrow.

Figure 5 depicts the network interaction of dysregulated miRNAs in tissue and their putative targets (predicted and validated) obtained after the gene set enrichment analysis (GSEA). The GSEA provided 376 putative targets. Twenty-one genes were identified as potential targets of more than one miRNA. *Thrombospondin 2* (*THBS2*) was the only predicted gene that could be targeted by three dysregulated miRNAs: miR-21-5p, miR-195-5p, and miR-144-3p.

**Figure 6:**
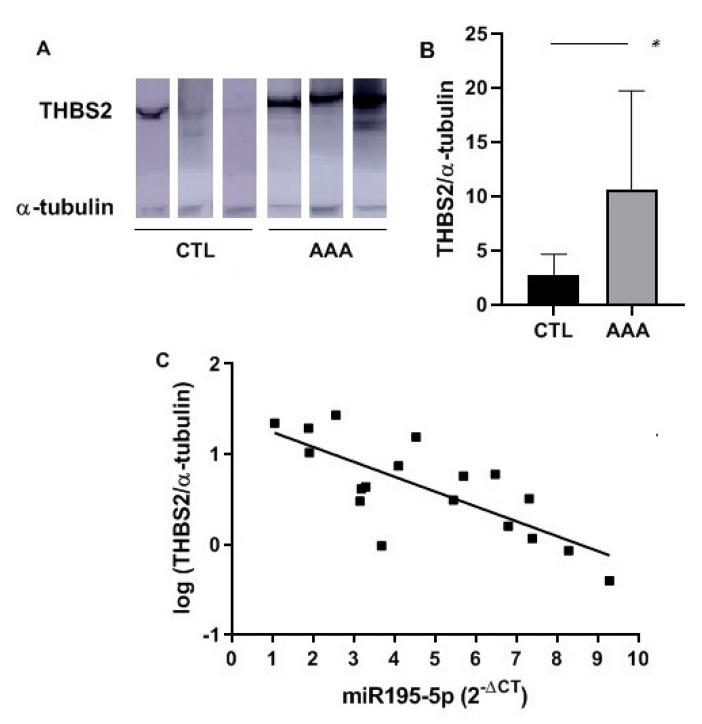
Expression levels of THBS2 in tissue samples of AAA patients and organ donors (CTL) quantified by Western Blot. (**A**) Bands obtained in Western blot analysis of THBS2 in AAA and CTL tissue samples (representative image of three samples of each group); (**B**) Bar graph of expression levels of THBS2 in tissue specimens of AAA (*n* = 11) and CTL (*n* = 7) group calculated as a relative expression using α-tubulin for normalization; (**C**) Correlation of log (THBS2/α-tubulin) levels and miRNA-195-5p (2^−∆Ct^) expression in tissue samples (*n* = 18, CTL and AAA). *, *p* < 0.05.

The 376 putative targets of the dysregulated miRNAs were reviewed. Five proteins, THBS2, LASP1, LAMB3, ITGAV, and COL11A1A, were selected according to their GO classification, the Kyoto Encyclopedia of Genes and Genomes (KEGG) [27] pathways in which they can be involved and their relationship with AAA or vascular disorders. Their expression level was quantified by Western blot in 18 tissue samples (11 patients and 7 organ donors). Levels of THBS2 were increased in AAA tissue samples compared with that of organ donors (*p* = 0.038; 95%CI, [0.497, 15.36]) (Figure 6A,B). Levels of ITGAV and LASP1 were also increased in AAA tissue but without significant differences. LAMB 3 and COL11A1 were undetectable in our tissue sample set. Remarkably, a negative correlation between THBS2 and miR-195-5p levels was found in tissue samples (ρ = −0.665, *p* = 0.0026) (Figure 6C).

## 3. Discussion

In the present study, we have found seven miRNAs (miR-103a-3p, miR-27b-3p, miR-99a-5p, miR-375, miR-221-3p, miR-146a-5p, and miR-1260) overexpressed in plasma of AAA patients compared with that of healthy volunteers. Two of these miRNAs, miR-27b-3p and miR-221-3p, remained significantly overexpressed after multivariate adjustment according to cardiovascular risk factors. These miRNAs were also slightly overexpressed in plasma of atherosclerotic patients reflecting a plausible participation in a common mechanism. In fact, Pereira da Silva et al. [28] recently highlighted that circulating miR-27b-3p and miR-221-3p have been defined as potential biomarkers of atherosclerosis disease and their role in plaque development has been previously described. Thus, the miR-27b family act as a mediator in angiogenesis, lipid metabolism, inflammatory and immune response, oxidative stress, and insulin resistance [29]. Otherwise, miR-221-3p regulates inflammation and angiogenesis by controlling endothelial cell migration, proliferation, and vascular smooth muscle cell (VSMC) growth [17,18,19]. We have found that plasma miR-27b-3p and miR-221-3p levels are higher in AAA than in severe atherosclerosis patients. According to these results, it is reasonable to think that they could be biomarkers of general vascular disorders, and their use in AAA disease, in combination with additional biomarkers, could be considered.

The studies about circulating miRNAs as biomarkers of AAA have been increasing in the last years. However, its application needs to be further elucidated. Kin et al. [9] found a different miRNAs’ expression pattern between AAA patients and healthy volunteers, which included miR-15a/b, miR-29, miR-124a, miR-126, miR-146a, miR-155, and miR-223. Zhang et al. [30] found that miR-191-3p, miR-455-3p, and miR-1281 were overexpressed in AAA patients. Stather et al. [12] observed a dysregulation in AAA patients for circulating let-7e, miR-15a, miR-196b, and miR-411, and they were also dysregulated in patients with peripheral artery disease (PAD). Recently, Spear et al. [31] found that levels of circulating miR-let-7f and miR-29b were elevated in AAA patients compared with PAD patients. In addition, only one study correlates circulating miRNAs with AAA growth [32]. In line with previous results, we have observed that those miRNAs that were elevated in AAA plasma were also slightly elevated in the plasma of severe atherosclerotic patients. This indicates that several biomarkers could be common for cardiovascular diseases. These results could be useful to elucidate common underlying mechanisms and to better understand the relationship between AAA and atherosclerosis. Despite the promising utility of miRNAs as AAA biomarkers, it is worth noting that, to date, the results depicted in different research studies do not coincide and seem difficult to replicate. These discrepancies among publications could be due to the variability of methodologies employed for miRNAs quantification; type of biofluid studied, and anticoagulant used during blood sampling, RNA isolation methods, qPCR reagents and conditions, or the normalization strategy employed [13]. Moreover, this lack of correlation could be enhanced by the fact that AAA is a multifactorial and heterogeneous disease with several comorbidities and variability among patients, and the perfect control group to compare the expression levels is also difficult to establish. To overcome these difficulties, we have selected an endogenous and stable miRNA among a group of candidates by RefFinder. To our knowledge, this is the only study about circulating miRNAs in AAA in which the election of the endogenous reference miRNA has been experimentally selected according to its stable expression among samples. In any case, the lack of reproducibility among publications emphasizes the importance of standardization in the methodology and protocols used, and the need for validation in independent populations.

We have also quantified 17 miRNAs in AAA and healthy arterial tissue. Six of them were downregulated (miR-27b-3p, miR-195-5p, miR-133b, miR-133a, miR-1, miR-29b-3p) and four were upregulated (miR103a-3p, miR-146a-5p, miR-144-3p, miR-21-5p) in surgical AAA specimens. Several authors have described molecular mechanisms in which some of these miRNAs could be involved. Thus, it has been suggested that miR-21-5p is increased in AAA as a physiological response to counteract the pathology and leads to a decrease in PTEN (phosphatase and tensin homolog) and subsequent survival of VSMCs [3]. Moreover, it is highly expressed in endothelial cells, and it is known to be regulated by shear stress [33]. The role of miR-29b-3p has been widely explored and controversial, but it is known to modulate the fibrotic response in the aortic wall through several collagen isoforms, as well as elastin [34]. Jiao et al. [16] observed in vitro and in a murine model that miR-103a-3p could be involved in AAA via targeting ADAM10. The function of miR-133a, miR-133b, and miR-1 has been widely studied in cardiovascular disorders and in the context of AAA, Pahl et al. [15] suggested their involvement through VSMC apoptosis. Mechanistically, miR-195 targets ECM (extracellular matrix) proteins and also MMPs (matrix metalloproteinases) [3]; moreover, it can regulate VSMC phenotype, inhibiting their proliferation and migration [35]. In our study, the biggest differences in expression levels were observed in miR-146a-5p and miR-144-3p. It is known that miR-146a-5p regulates inflammatory processes through cytokines. After a biochemical stimulus (TNF-α), the expression of miR-146a-5p is induced, regulating TRAF 6 or IRAK 1 [36,37,38]. Moreover, this miRNA can also regulate phenotypical changes in macrophages (M1/M2) and mediate lipid transportation and cytokines liberation in macrophages [39], and it has also been related to VSMC migration and proliferation [18]. Recently, Zhang et al. suggested that miR-146a regulates inflammation in AAA via CARD10 [40]. Furthermore, the overexpression of miR-146a-5p in AAA tissue samples has been previously described [9,15,40,41]. Conversely, only one recent study explored the levels of miR-144-3p in AAA tissue [42], and they postulated their relationship with diabetes. Moreover, miR-144-3p regulates ABCA1 in macrophages, and it can be involved in plaque formation through impairing reverse cholesterol transport and promoting pro-inflammatory cytokine production [43]. Thus, the possible role of miR-144-3p in AAA development needs to be further investigated.

Similar to what occurred in plasma, the results in tissue samples differ among studies. As previously reported [10,15,23,44,45], we have observed a downregulation of miR-133b, miR-29b-3p, and miR-27b-3p in AAA tissue, whereas other authors found them upregulated [9,46]. In the case of miR-21-5p, most studies report an increase in AAA tissue [9,15,22], while only one group found a decrease [21].

Surprisingly, although we found that miR-221-3p and miR-27b-3p were upregulated in plasma, they were not upregulated in tissue. While no differences in miR-221-3p expression in tissue were observed, miR-27b-3p was significantly downregulated in surgical AAA specimens. Other authors have also seen discrepancies between plasma and tissue expression levels [9,12], this indicates that further studies in paired plasma/tissue samples are needed to elucidate the existence of a correlation between systemic and localized miRNA expression.

We have explored the putative targets controlled by the dysregulated miRNAs in tissue samples in silico as well as the main BP, MF, and CC affected according to GO. The extracellular matrix and the basement membrane, which is known to play a relevant role in AAA development [47,48], were found among the main CC. Otherwise, ERK1/2 cascade, response to a mechanical stimulus or regulation of lipid, ROS, and carbohydrate metabolic processes were some of the BP in which these miRNAs could be involved. These processes are related directly or indirectly to AAA [49,50,51]. Regarding their targets after GSEA, we observed that some genes can be targeted by more than one miRNA dysregulated in tissue. In particular, *THBS2* is the only gene that could be regulated by three of the dysregulated miRNAs. It is worth noting that some of the predicted targets encode proteins that could be related to vascular processes. Thus, we selected them for their quantification in tissue samples. LASP1 is an actin-binding cytoskeletal protein involved in cell migration and chemotaxis [52]. ITGAV belongs to an extracellular matrix receptor protein family related to angiogenesis and to AAA [53]. LAMB3 is an extracellular matrix protein that belongs to the laminin family, and their relationship with thoracic aneurysm has been previously studied [54], and COL11A1 is a minor fibrillar collagen, structurally and biologically related to collagen type V [55]. Interestingly, THBS2 is an extracellular matrix glycoprotein that regulates a variety of cell–matrix interactions. It belongs to the Thrombospondin family, which is known to play an important role in cardiovascular diseases [56], although THBS1 is the best-known member and THBS2 itself has not been deeply studied. It is thought to modulate cell adhesion in fibroblasts by regulating the availability of MMP2 in the extracellular matrix [57], and it is able to promote MMP13 expression and cell migration [58]. Moreover, a recent in vitro study has demonstrated that THBS2 also induces VSMC proliferation [59]. THBS2 has been previously related to cardiovascular mortality in AAA patients [60], and even a previous study related a polymorphism in the *THBS2* gene with thoracic aortic aneurysm [61]. To our knowledge, this is the first study in which an overexpression of THBS2 has been described in AAA human tissue. Moreover, a negative correlation has been observed between THBS2 and miR-195-5p levels, suggesting that THBS2 could be a real target of miRNA 195-5p. In fact, THBS2 and miR-195-5p could exert different effects on VSMC, inducing or inhibiting their proliferation, respectively [35,59]. Future in vitro studies in cell cultures and in vivo studies in animal models will further verify the regulation of THBS2 by miRNA-195-5p and will provide further insights on the molecular mechanisms involved.

The main limitation of our study is that plasma and tissue samples do not correspond to the same patients because tissue samples belong to a pre-existing collection stored at La Fe Biobank and most plasma samples belonged to patients that underwent endovascular repair; thus, tissue samples were not available from these patients. Since non-paired plasma and tissue samples from AAA patients were studied, results should be taken cautiously. Additional studies in paired tissue and plasma samples are required to corroborate our results. In addition, the results obtained from tissue could not be adjusted in a multivariable model since, to guarantee confidentiality, we could not obtain clinical characteristics of the organ donors studied. It is very likely that the small sample size of the organ donor cohort, in conjunction with potential sampling biases, makes the cohort an imperfect control for AAA tissue comparison. There remains a danger of overinterpretation of these data, particularly the GSEA results. To circumvent this problem, different tissue sources have been previously suggested as controls [8,9,15]; however, their use also implies several limitations. Our case-control study includes patients that had already been diagnosed with AAA at inclusion. Therefore, we cannot ensure whether the dysregulation of these miRNAs is a cause or a consequence of AAA development. Prospective population-based studies would definitively clarify this issue. However, these studies require the recruitment of a tremendous number of individuals and long-term follow up. Additionally, our study was performed in a relatively small set of samples, especially the screening stage, making it difficult to perform a robust predictive model. The validation of our results in a larger independent cohort of AAA patients would definitively reinforce our findings.

## 4. Materials and Methods

### 4.1. Patients

For the plasma study, 57 male subjects were consecutively recruited, 30 patients objectively diagnosed with large AAA by means of computed tomography (indication for surgery: aortic diameter >5.5 cm) undergoing open surgery (*n* = 10) or endovascular (*n* = 20) repair (AAA group), 16 healthy volunteers (CTL group) and 11 patients with severe atherosclerosis who underwent carotid endarterectomy (CE group). The presence of AAA was ruled out in CTL and CE group by ultrasound scanning. Patients with genetic syndromes (Marfan or Ehler-Danlos), active malignant diseases, autoimmune or systemic inflammatory disease (cancer, acquired immunodeficiency syndrome, or hepatitis) were excluded. Cardiovascular risk factors, DM, DL, and HTN, were previously diagnosed by primary care medical doctors according to guidelines (European Society of Cardiology, American Diabetes Association). Patients’ medical records were reviewed, and these cardiovascular risk factors were considered positive when diagnosed, even if they were controlled by medication at sampling. Smoking was considered positive if subjects were current or former smokers within the last 5 years since the cardiovascular risk is reduced after this period [62].

Additionally, surgical specimens were obtained from 29 independent subjects: 21 patients objectively diagnosed with AAA that underwent open surgery (CE) and 8 organ donors without AAA evidence. Organ donors were subjects suitable for transplantation (from healthy brain-dead patients), thus, they were considered healthy regarding malignant and infectious diseases. Only visually healthy arteries were accepted. Data from organ donors were impossible to obtain due to confidentiality and anonymity hospital policy related to donations.

The present study was performed according to the declaration of Helsinki and was approved by the ethical committee of the Medical Research Institute Hospital La Fe (reference 2013/0068, approved on 9 April 2013, and revised on 7 July 2015). All participants agreed to participate and gave informed consent before the investigation.

### 4.2. Sample Collection

Peripheral blood was collected in 3 mL tubes with 5.4 mg of K_2_EDTA as an anticoagulant. Samples were processed within 3 h after collection. Tubes were centrifuged at 1811× *g* for 30 min at 4 °C. Plasma was recovered, dispensed in aliquots of 250 µL and stored at −80 °C until used.

An independent set of abdominal aortic samples were collected during open repair surgery (patients) and during organ extraction procedure (organ donors, ischemia time <2 h). Samples were washed with phosphate-buffered saline pH 7.4, and immediately snap-frozen in liquid nitrogen. Tissue samples were stored in liquid nitrogen at the La Fe Biobank (La Fe University and Polytechnic Hospital) until used.

### 4.3. RNA Isolation and cDNA Synthesis from Plasma and Tissue Samples

Total RNA was isolated from 250 µL plasma using the miRNeasy mini kit (Qiagen, Hilden, Germany) according to an optimized method previously reported [13]. Briefly, plasma aliquots were centrifuged for 5 min, at 4 °C and 3000× *g* to eliminate cellular debris. Two hundred microliters of plasma supernatant was transferred to a tube with 1 mL of Qiazol (Qiagen, Hilden, Germany), 1 µL of carrier (1 µg/µL yeast RNA, Invitrogen, ThermoFisher Scientific, Waltham, MA, USA) and 1 µL of spike in the mix (UniSp2/4/5, Exiqon, Vedbaek, Denmark), and mixed gently. After a 5 min incubation at room temperature, 200 µL of chloroform was added, and centrifuged at 12,000× *g*, 15 min at 4 °C to allow phase separation. Ethanol in a proportion of 1.5:1 (volume:volume) was added to the liquid phase followed by 4 cleaning steps with the buffers supplied in the kit. RNA was finally eluted in 30 µL of DNase/RNase-free sterile distilled water.

Total RNA and protein fraction were simultaneously isolated from tissue samples with the miRVANA PARIS^®^ kit (Invitrogen, Thermo Fisher Scientific, Waltham, MA, USA) according to the manufacturer´s instructions. Briefly, a cross-section of tissue sample was homogenized with the TissueLyser LT^®^ (Qiagen, Hilden, Germany) (homogenization conditions: 2 beads, 50 mHz, 5 min) in 625 µL of cold cell disruption buffer with 7 µL of proteases inhibitor cocktail (Calbiochem, Merk, Darmstadt, Germany). Four hundred microliters of tissue lysate were used for RNA isolation and the remaining volume for protein isolation. RNA isolation was carried out, adding 400 µL denaturing solution provided in the kit, followed by phase separation with 800 µL of phenol-chloroform. After the washing steps indicated in the protocol, RNA was eluted with 100 µL of DNase/RNase-free sterile distilled water pre-heated at 95 °C. The protein fraction was sonicated (3 pulses of 5 s), distributed in 50 µL aliquots, snap-frozen in liquid N_2_, and stored at −80 °C until use. RNA concentration (A260) and purity (A280/A260) were determined by spectrophotometry in a Nanodrop ND100 (Thermo Fisher Scientific, Waltham, MA, USA). In our samples, A280/260 = 1.996 ± 0.107 (mean ± std deviation). Due to RNA quantity was variable among samples; a working solution at 5 ng/µL was prepared for each sample.

For the screening stage, cDNA was obtained from 5 µL of plasma RNA with the Universal cDNA synthesis kit^®^ (Exiqon, Vedbaek, Denmark) according to the supplied protocol (final volume 25 µL). Due to the use of an RNA carrier during the isolation, the final RNA yield included the RNA isolated from plasma plus the carrier RNA. Therefore, plasma RNA retrotranscription was based on volume (µL) rather than RNA quantity (ng), according to suppliers’ recommendations. To quantify the expression level of selected miRNAs, cDNA was obtained from 10 ng of total tissue RNA or from 2 µL of plasma RNA using the same technology (final reaction volume 10 µL). Reaction mix containing RNA, enzyme, buffer, RNAse-free water, and UniSp6 RNA spike in the template, was incubated 60 min at 42 °C followed by 5 min at 95 °C for reverse transcriptase inactivation. Reactions were carried out in a thermocycler TC-412 (Techne, Staffordshire, UK). Two independent reactions were performed for each sample as replicates.

### 4.4. miRNA Quantification

A subset of 7 samples of plasma cDNA from AAA patients and 7 from healthy volunteers was included at the screening stage. A total of 179 miRNAs were quantified using a predesigned Serum/plasma Focus microRNA PCR Panel V4 (Exiqon) by quantitative Real-Time Polymerase Chain Reaction (RT-qPCR) and with ExiLENT SYBR Green^®^ master mix (Exiqon Vedbaek, Denmark) as a fluorophore, according to the manufacturer´s indications. Briefly, cDNA (dilution 1/40), water, and PCR master Mix (which includes Sybr Green) were added to a 384-well PCR plate supplied that includes the LNA™ primer sets. qPCR reactions were performed as follows: a polymerase activation/denaturation cycle of 10 min at 95 °C, followed by 45 cycles of 10 s at 95 °C and 1 min at 60 °C, with a ramp-rate of 1.6 °C/s. All reactions were carried out in a LightCycler 480 II (Roche, Mannheim, Germany).

From the screening stage, 10 miRNAs (miR-27b-3p [MIMAT0000419], miR-152-3p [MIMAT0000438], miR-7-1-3p [MIMAT0004553], miR-375 [MIMAT0000728], miR-103a-3p [MIMAT0000101], miR-144-3p [MIMAT0000436], miR-146a-5p [MIMAT0000449], miR-1260a [MIMAT0005911], miR-221-3p [MIMAT0000278], and miR-99a-5p [MIMAT0000097]) were selected as dysregulated in AAA plasma. Thus, they were quantified in the whole plasma sample set (N = 57), together with miR-195-5p [MIMAT0000461] because of its relation to AAA in previous literature [20,30,63]. miR-191-5 [MIMAT0000440] was used as normalizer. miRNAs were quantified using the LNA^®^ primer set and ExiLENT SYBR Green^®^ master mix (Exiqon, Vedbaek, Denmark). In tissue specimens, these 11 miRNAs with 6 additional miRNAs (miR-1 [MIMAT0000416], miR-133a [MIMAT0000427], miR-133b [MIMAT0000770], miR-21-5p [MIMAT0000076], miR29b-3p [MIMAT0000100], and miR-155-5p [MIMAT0000646]) selected according to bibliography [9,15,21,22], and miR423-5p [MIMAT0004748] as normalizer, were quantified in 21 samples from AAA tissue and 8 samples from healthy tissue from organ donors, using the same technology. RT-qPCR reactions were carried out in 96-well plates under the same conditions previously described (cDNA dilution: 1/40 for plasma samples and 1/80 for tissue samples).

### 4.5. Identification of miRNA Targets

Putative target genes of significantly dysregulated miRNAs in tissue samples were estimated by calculating a regulation score on each target, as previously described [64], and applying a gene set enrichment analysis (GSEA; a computational method that allows to assess whether a subset of genes shows significant over-representation related to a defined characteristic). Briefly, target genes were obtained using a computational analysis that compiles the information allocated in the following databases: TargetScan (conserved site context scores, version 7.1), miRDB (release 5.0), and validated information from miRTarBase (version 7.0). The analysis was based on a prediction blast with an e-value of 10^−5^. A weight of 0.9 was applied. This search renders two types of results, validated targets (when previous studies have experimentally validated this regulation) and predicted targets (those being theoretically estimated based on the free binding energy between a miRNA and a putative target mRNA sequence). Gene Set Enrichment Analysis was performed by ranking all target genes taking into account the fold-change values of each individual miRNA. Following the procedure previously described [65], the biological processes (BP), molecular functions (MF), and cellular components (CC) affected according to the Gene Ontology (GO) were obtained. According to GO definitions, MF terms describe activities that occur at the molecular level; CC refers to the locations relative to cellular structures in which a gene product performs a function, either cellular compartments or stable macromolecular complexes of which they are parts, and BP defines larger processes accomplished by multiple molecular activities.

### 4.6. Western Blot Analysis of Target Proteins

Total protein concentration was quantified with the Pierce™ BCA protein assay kit (Thermo Fisher Scientific, Waltham, MA, USA) and compared to a standard curve of bovine serum albumin, according to the manufacturer’s instructions. Twenty micrograms of protein per lane were subjected to 4%–12% SDS-polyacrylamide gel electrophoresis (PAGE) under reducing conditions and transferred to a PVDF (polyvinylidene difluoride) membrane (10 V, 25 min) using the iBlot Gel Transfer (Life Technologies, Carlsbad, CA, USA). Membranes were blocked for 1 h at room temperature with 5% non-fat milk in Tris-Buffer solution containing 0.1% Tween-20, and then incubated overnight at 4 °C with specific antibodies in blocking buffer, followed by a 1 h incubation with horseradish peroxidase-conjugated secondary antibody (dilution 1:3000). Finally, bands were detected by chemiluminescence using ECL (Ge HealthCare, Amersham UK) in an Amersham Imager 600 (GE HealthCare, Amersham, UK). Primary specific antibodies were ITGAV (Integrin α5), dilution 1:1000, LASP1 (LIM and SH3 domain protein 1), dilution 1:4000, and LAMB3 (Laminin 3β subunit), dilution 1:1000, rabbit polyclonal antibodies (Proteintech, Rosemont, IL, USA); COL11A1 (Collagen XI α1), dilution 1:500, and TSP-2 (THBS2), dilution 1:1000, rabbit polyclonal antibodies (GeneTex, Hsinchu City, Taiwan), and α-tubulin mouse monoclonal (Cell Signalling, Leiden, The Netherlands) as reference. Secondary antibodies were ECL™ labeled anti-mouse IgG, HRP-linked F(ab’)_2_ fragment from sheep (GE Healthcare, Amersham, UK) and goat anti-rabbit IgG (H + L)-HRP Conjugate (Bio Rad, Hercules, CA, USA).

### 4.7. Data Analysis

The results from RT-qPCR expression (CTs) were normalized using the endogenous and most stable miRNA in plasma or tissue analyses, selected using the RefFinder software [14] following previously stated strategies [66,67]. This software comprises the results of 4 commonly employed algorithms: Delta CT, BestKeeper, Normfinder, and Genorm. miRNA expression levels were calculated for each sample as 2^−ΔCT^ against the selected reference miRNA. Fold-change is defined as the ratio of the average expression level of a miRNA in patients and controls. After the screening stage in plasma of AAA patients and healthy controls, the most relevant miRNAs and clinical variables were identified using a classification algorithm of Random Forest (R package) [68]. In the analysis, 144 variables were included: 139 miRNAs, age, HTN, DM, DL, and smoking habits (mtree = 500). The final discriminant miRNAs were then selected considering their expression level, fold-change, and relevance in cardiovascular processes. Continuous variables were summarized as mean (standard deviation) and compared between 2 groups with the Student *t*-test. Categorical variables were expressed as absolute and relative frequencies and compared with the Fisher´s test. In the plasma study, the expression level of each miRNA was compared among the different groups studied (AAA, CTL, and CE) with a multivariable linear regression model, including cardiovascular risk factors (age, HTN, DL, DM, and smoking) as confounding variables. In the tissue study, the expression levels of each miRNA were compared between groups (AAA and organ donors) with a linear regression model. Correlations were analyzed with the Pearson Correlation test. The analysis was performed using the statistical software R (3.5.0 version, Vienna, Austria) and GraphPad Prism v.8 (San Diego, CA, USA), and a *p*-value < 0.05 was considered statistically significant. Cytoscape™ version 3.6.0, a free software for integrated models of biomolecular interaction networks [69], was used to generate a network showing the miRNA–mRNA connections. miRNAs were represented as circles, and target mRNAs were represented as squares.

## 5. Conclusions

In conclusion, miR-27b-3p and miR-221-3p were overexpressed in plasma of AAA patients independently of the classical cardiovascular risk factors. Furthermore, we have confirmed the dysregulation of 10 miRNAs in AAA tissue samples: miR-27b-3p, miR-195-5p, miR-133b, miR-133a, miR-1, miR-29b-3p, miR-103a-3p, miR-146a-5p, miR-144-3p, and miR-21-5p. In line with previous publications, our results reflect the importance of miRNAs in AAA development, and the dysregulated miRNAs that we describe herein may be reliable biomarkers of the disease. Our in silico study revealed new putative targets of the dysregulated miRNAs in AAA. In fact, the overexpression of one of those putative targets, THBS2, was confirmed in AAA tissue. Moreover, a negative correlation was observed between THBS2 levels and that of miR-195-5p, one of its regulatory miRNAs. These results point out that THBS2 could be a real target of miR-195-5p, and both could be involved in a common pathway related to AAA.

## Figures and Tables

**Table 1:** Clinical characteristics of the individuals included in the study.

Variable	CTL	AAA	CE
	Participants Included in the Screening Stage of the Plasma Study		
	*n* = 7	*n* = 7	-
Sex (male)	7 (100.0%)	7 (100.0%)	-
Age (SD)	62 (3)	72 (7)	-
HTN	5 (71.4%)	7 (100.0%)	-
DM	2 (28.6%)	2 (28.6%)	-
DL	5 (71.4%)	6 (85.7%)	-
Smoking *	4 (57.1%)	2 (28.6%)	-
	**Participants included in the complete plasma study**		
	***n* = 16**	***n* = 30**	***n* = 11**
Sex (male)	16 (100.0%)	30 (100.0%)	11 (100.0%)
Age (SD)	63 (5)	74 (8)	68 (6)
HTN	10 (62.5%)	27 (90.0%)	7 (63.6%)
DM	2 (12.5%)	13 (43.3%)	6 (54.5%)
DL	14 (87.5%)	20 (66.7%)	7 (63.6%)
Smoking *	4 (25.0%)	14 (46.7%)	8 (72.7%)
Open Surgery	-	10 (33.3%)	-
Aneurysm Diameter, mm (SD)	-	57.3 (10.3)	-
Statin use	4 (25.0%)	21 (70.0%)	10 (90.9%)
Anti-hypertensives (no beta-blockers)	10 (62.5%)	23 (76.7%)	7 (63.6%)
Anti-hypertensives (beta-blockers)	2 (12.5%)	8 (26.7%)	4 (36.4%)
ASA	2 (12.5%)	11 (36.7%)	7 (63.6%)
Other antiplatelet therapies	1 (6.3%)	5 (16.7%)	3 (27.3%)
Anti-coagulants	0 (0.0%)	4 (13.3%)	1 (9.1%)
	**Participants included in the complete tissue study**		
	***n* = 8**	***n* = 21**	**-**
Sex (male)	-	20 (95.2%)	-
Age (SD)	-	65.3 (6.5)	-
HTN	-	17 (81.0%)	-
DM	-	10 (47.6%)	-
DL	-	13 (61.9%)	-
Smoking *	-	10 (47.6%)	-
Open Surgery	-	21 (100.0%)	-
Aneurysm Diameter, mm (SD)	-	61.7 (10.9)	-
Statin use	-	14 (66.7%)	-
Anti-hypertensives (no beta-blockers)	-	16 (76.2%)	-
Anti-hypertensives (beta-blockers)	-	6 (28.6%)	-
ASA	-	4 (19.0%)	-
Other antiplatelet therapies	-	3 (14.3%)	-
Anti-coagulants	-	3 (14.3%)	-

CTL, healthy volunteers; AAA, patients with an abdominal aortic aneurysm; CE, patients with severe atherosclerosis who underwent carotid endarterectomy; HTN, hypertension; DM, diabetes mellitus; DL, dyslipidemia; ASA, acetyl salicylic acid; *, Current smokers or former smokers within last 5 years.

**Table 2:** Fold-change of the plasma microRNAs (miRNAs) included in the first 30 variables rendered by Random Forest. Fold-change is defined as the ratio of the average expression level of a miRNA in abdominal aortic aneurysm (AAA) and control (CTL). Expression levels are calculated by the 2^−ΔCt^ method. Negative fold-change values are calculated as –(1/Fold-change).

miRNA Name	Fold-Change (AAA/CTL)
miR-27b-3p	1.39
miR-339-3p	1.92
let-7g-5p	1.10
miR-152-3p	2.52
miR-7-1-3p	−2.27
miR-375	2.45
miR-103a-3p	−1.39
miR-193a-5p	3.44
miR-154-5p	1.01
miR-326	2.59
miR-362-3p	−1.37
miR-140-3p	1.45
miR-144-3p	−2.27
miR-146a-5p	1.66
miR-130b-3p	1.51
miR-1260a	2.06
miR-29a-3p	1.34
miR-144-5p	1.23
miR-100-5p	1.65
miR-106b-5p	−1.27
miR-766-3p	1.73
miR-125a-5p	1.42
miR-142-5p	1.23
miR-22-3p	1.96
miR-221-3p	1.50
miR-99a-5p	2.71
miR-30e-3p	1.81
let-7b-3p	3.24
miR-125b-5p	1.34

**Table 3:** Expression levels of the miRNAs quantified in the AAA and CTL plasma sample collections. Fold-change is defined as the ratio of the average expression level of a miRNA in AAA and CTL. Expression levels are calculated by the 2^−ΔCT^ method. *p*-values were calculated after adjustment with a multivariable model including age, hypertension (HTN), diabetes mellitus (DM), dyslipidemia (DL), and smoking habits.

miRNA Name	miRNA Sequence	Fold-Change (AAA vs. CTL)	*p*-Value	Estimate	Lower 95%	Upper 95%
miR-152-3p	ucagugcaugacagaacuugg	1.5	0.357	0.028	−0.032	0.087
miR-144-3p	uacaguauagaugauguacu	1	0.804	−0.230	−2.081	1.621
miR-27b-3p	uucacaguggcuaaguucugc	1.6	0.043	0.427	0.014	0.841
miR-103a-3p	agcagcauuguacagggcuauga	1	0.284	0.528	−0.452	1.509
miR-99a-5p	aacccguagauccgaucuugug	1.9	0.183	0.081	−0.04	0.201
miR-375	uuuguucguucggcucgcguga	2	0.139	0.075	−0.025	0.174
miR-221-3p	agcuacauugucugcuggguuuc	1.9	0.001	0.333	0.145	0.521
miR-146a-5p	ugagaacugaauuccauggguu	1.6	0.457	0.054	−0.09	0.198
miR-1260	aucccaccucugccacca	2	0.238	0.049	−0.034	0.132

**Table 4:** Expression levels of the miRNAs quantified in the tissue sample collection of AAA patients and CTL. Values were normalized with miR-423-5p by the 2^−ΔCt^ method. Fold-change is defined as the ratio of the average expression level of a miRNA in patients and controls. Negative fold-change values were calculated as –(1/Fold-change). *p*-values were calculated after adjustment with a linear regression model.

miRNA Name	miRNA Sequence	Fold-Change (AAA vs. CTL)	*p*-Value	Estimate	Lower 95%	Upper 95%
miR-221-3p	agcuacauugucugcuggguuuc	1.1	0.742	0.171	−0.885	1.226
miR-27b-3p	uucacaguggcuaaguucugc	−2.0	<0.001	−15.244	−22.319	−8.169
miR-99a-5p	aacccguagauccgaucuugug	1.4	0.277	1.947	−1.653	5.548
miR-103a-3p	agcagcauuguacagggcuauga	1.3	0.050	3.856	0.007	7.705
miR-146a-5p	ugagaacugaauuccauggguu	5.8	<0.001	1.251	0.662	1.840
miR-1260	aucccaccucugccacca	1.2	0.677	0.379	−1.464	2.221
miR-144-3p	uacaguauagaugauguacu	7.2	0.001	19.002	8.656	29.348
miR-152-3p	ucagugcaugacagaacuugg	1.1	0.380	0.246	−0.320	0.813
miR-195-5p	uagcagcacagaaauauuggc	−1.5	0.023	−2.248	−4.163	−0.333
miR-155-5p	uuaaugcuaaucgugauagggguu	1.9	0.082	0.316	−0.043	0.676
miR-21-5p	uagcuuaucagacugauguuga	1.9	0.012	86.025	20.360	151.689
miR-133b	uuugguccccuucaaccagcua	−4.6	<0.001	−2.82	−3.637	−2.002
miR-133a	uuugguccccuucaaccagcug	−4.4	<0.001	−1.643	−2.150	−1.135
miR-1	uggaauguaaagaaguauguau	−4.4	<0.001	−1.628	−2.105	−1.150
miR-29b-3p	uagcaccauuugaaaucaguguu	−1.4	0.018	−3.509	−6.373	−0.645

## Supplementary Materials

Supplementary materials can be found at https://www.mdpi.com/1422-0067/21/13/4600/s1.

## Abbreviations

AAA	Abdominal aortic aneurysm
AUC	Area under curve
ASA	Acetyl salicylic acid
BP	Biological processes
CE	Carotid endarterectomy
CC	Cellular components
COL11A1A	Collagen XI α1
CT	Cycle threshold
CTL	Control
DM	Diabetes mellitus
DL	Dyslipidemia
ECM	Extracellular matrix
GO	Gene Ontology
GSEA	Gene set enrichment analysis
HTN	Hypertension
ITGAV	Integrin α5
LAMB3	Laminin 3β subunit
LASP1	LIM and SH3 domain protein 1
miRNA	microRNA
MF	Molecular function
MMP	Matrix Metalloproteinases
THBS2	Thrombospondin 2
VSMC	Vascular smooth muscle cells

## References

[1] Cornuz J., Sidoti Pinto C., Tevaearai H., Egger M. (2004). Risk factors for asymptomatic abdominal aortic aneurysm: Systematic review and meta-analysis of population-based screening studies. Eur. J. Public Health.

[2] Sakalihasan N., Limet R., Defawe O.D. (2005). Abdominal aortic aneurysm. Lancet.

[3] Kumar S., Boon R.A., Maegdefessel L., Dimmeler S., Jo H. (2019). Role of Noncoding RNAs in the Pathogenesis of Abdominal Aortic Aneurysm. Circ. Res..

[4] Ambros V. (2004). The functions of animal microRNAs. Nature.

[5] Zorio E., Medina P., Rueda J., Millan J.M., Arnau M.A., Beneyto M., Marin F., Gimeno J.R., Osca J., Salvador A. (2009). Insights into the role of microRNAs in cardiac diseases: From biological signalling to therapeutic targets. Cardiovasc. Hematol. Agents Med. Chem..

[6] Faruq O., Vecchione A. (2015). microRNA: Diagnostic Perspective. Front. Med..

[7] Raffort J., Lareyre F., Clement M., Mallat Z. (2016). Micro-RNAs in abdominal aortic aneurysms: Insights from animal models and relevance to human disease. Cardiovasc. Res..

[8] Biros E., Moran C.S., Wang Y., Walker P.J., Cardinal J., Golledge J. (2014). microRNA profiling in patients with abdominal aortic aneurysms: The significance of miR-155. Clin. Sci. (Lond.).

[9] Kin K., Miyagawa S., Fukushima S., Shirakawa Y., Torikai K., Shimamura K., Daimon T., Kawahara Y., Kuratani T., Sawa Y. (2012). Tissue- and plasma-specific MicroRNA signatures for atherosclerotic abdominal aortic aneurysm. J. Am. Heart Assoc..

[10] Maegdefessel L., Spin J.M., Raaz U., Eken S.M., Toh R., Azuma J., Adam M., Nakagami F., Nagakami F., Heymann H.M. (2014). miR-24 limits aortic vascular inflammation and murine abdominal aneurysm development. Nat. Commun..

[11] Spear R., Boytard L., Blervaque R., Chwastyniak M., Hot D., Vanhoutte J., Staels B., Lemoine Y., Lamblin N., Pruvot F.R. (2015). Adventitial Tertiary Lymphoid Organs as Potential Source of MicroRNA Biomarkers for Abdominal Aortic Aneurysm. Int. J. Mol. Sci..

[12] Stather P.W., Sylvius N., Sidloff D.A., Dattani N., Verissimo A., Wild J.B., Butt H.Z., Choke E., Sayers R.D., Bown M.J. (2015). Identification of microRNAs associated with abdominal aortic aneurysms and peripheral arterial disease. Br. J. Surg..

[13] Ramón-Núñez L.A., Martos L., Fernández-Pardo Á., Oto J., Medina P., España F., Navarro S. (2017). Comparison of protocols and RNA carriers for plasma miRNA isolation. Unraveling RNA carrier influence on miRNA isolation. PLoS ONE.

[14] Xie F., Xiao P., Chen D., Xu L., Zhang B. (2012). miRDeepFinder: A miRNA analysis tool for deep sequencing of plant small RNAs. Plant. Mol. Biol..

[15] Pahl M.C., Derr K., Gabel G., Hinterseher I., Elmore J.R., Schworer C.M., Peeler T.C., Franklin D.P., Gray J.L., Carey D.J. (2012). MicroRNA expression signature in human abdominal aortic aneurysms. BMC Med. Genomics.

[16] Jiao T., Yao Y., Zhang B., Hao D.C., Sun Q.F., Li J.B., Yuan C., Jing B., Wang Y.P., Wang H.Y. (2017). Role of MicroRNA-103a Targeting ADAM10 in Abdominal Aortic Aneurysm. BioMed. Res. Int..

[17] Li Y., Song Y.H., Li F., Yang T., Lu Y.W., Geng Y.J. (2009). MicroRNA-221 regulates high glucose-induced endothelial dysfunction. Biochem. Biophys. Res. Commun..

[18] Chen L.J., Lim S.H., Yeh Y.T., Lien S.C., Chiu J.J. (2012). Roles of microRNAs in atherosclerosis and restenosis. J. Biomed. Sci..

[19] Zhang X., Shao S., Geng H., Yu Y., Wang C., Liu Z., Yu C., Jiang X., Deng Y., Gao L. (2014). Expression profiles of six circulating microRNAs critical to atherosclerosis in patients with subclinical hypothyroidism: A clinical study. J. Clin. Endocrinol. Metab..

[20] Zampetaki A., Attia R., Mayr U., Gomes R.S., Phinikaridou A., Yin X., Langley S.R., Willeit P., Lu R., Fanshawe B. (2014). Role of miR-195 in aortic aneurysmal disease. Circ. Res..

[21] Busch A., Busch M., Scholz C.J., Kellersmann R., Otto C., Chernogubova E., Maegdefessel L., Zernecke A., Lorenz U. (2016). Aneurysm miRNA Signature Differs, Depending on Disease Localization and Morphology. Int. J. Mol. Sci..

[22] Maegdefessel L., Azuma J., Toh R., Deng A., Merk D.R., Raiesdana A., Leeper N.J., Raaz U., Schoelmerich A.M., McConnell M.V. (2012). MicroRNA-21 blocks abdominal aortic aneurysm development and nicotine-augmented expansion. Sci. Trans. Med..

[23] Maegdefessel L., Azuma J., Toh R., Merk D.R., Deng A., Chin J.T., Raaz U., Schoelmerich A.M., Raiesdana A., Leeper N.J. (2012). Inhibition of microRNA-29b reduces murine abdominal aortic aneurysm development. J. Clin. Investig..

[24] Iyer V., Rowbotham S., Biros E., Bingley J., Golledge J. (2017). A systematic review investigating the association of microRNAs with human abdominal aortic aneurysms. Atherosclerosis.

[25] Gene Ontology Consortium (2019). The Gene Ontology Resource: 20 years and still GOing strong. Nucleic Acids Res..

[26] Ashburner M., Ball C.A., Blake J.A., Botstein D., Butler H., Cherry J.M., Davis A.P., Dolinski K., Dwight S.S., Eppig J.T. (2000). Gene ontology: Tool for the unification of biology. The Gene Ontology Consortium. Nat. Genet..

[27] Kanehisa M., Goto S. (2000). KEGG: Kyoto encyclopedia of genes and genomes. Nucleic Acids Res..

[28] Pereira-da-Silva T., Coutinho Cruz M., Carrusca C., Cruz Ferreira R., Napoleão P., Mota Carmo M. (2018). Circulating microRNA profiles in different arterial territories of stable atherosclerotic disease: A systematic review. Am. J. Cardiovasc. Dis..

[29] Chen W.J., Yin K., Zhao G.J., Fu Y.C., Tang C.K. (2012). The magic and mystery of microRNA-27 in atherosclerosis. Atherosclerosis.

[30] Zhang W., Shang T., Huang C., Yu T., Liu C., Qiao T., Huang D., Liu Z. (2015). Plasma microRNAs serve as potential biomarkers for abdominal aortic aneurysm. Clin. Biochem..

[31] Spear R., Boytard L., Blervaque R., Chwastyniak M., Hot D., Vanhoutte J., Lamblin N., Amouyel P., Pinet F. (2019). Let-7f: A New Potential Circulating Biomarker Identified by miRNA Profiling of Cells Isolated from Human Abdominal Aortic Aneurysm. Int. J. Mol. Sci..

[32] Wanhainen A., Mani K., Vorkapic E., De Basso R., Bjorck M., Lanne T., Wagsater D. (2017). Screening of circulating microRNA biomarkers for prevalence of abdominal aortic aneurysm and aneurysm growth. Atherosclerosis.

[33] Wei Y., Schober A., Weber C. (2013). Pathogenic arterial remodeling: The good and bad of microRNAs. Am. J. Physiol. Heart Circ. Physiol..

[34] Maegdefessel L., Spin J.M., Adam M., Raaz U., Toh R., Nakagami F., Tsao P.S. (2013). Micromanaging abdominal aortic aneurysms. Int. J. Mol. Sci..

[35] Wang Y.S., Wang H.Y., Liao Y.C., Tsai P.C., Chen K.C., Cheng H.Y., Lin R.T., Juo S.H. (2012). MicroRNA-195 regulates vascular smooth muscle cell phenotype and prevents neointimal formation. Cardiovasc. Res..

[36] Cheng H.S., Sivachandran N., Lau A., Boudreau E., Zhao J.L., Baltimore D., Delgado-Olguin P., Cybulsky M.I., Fish J.E. (2013). MicroRNA-146 represses endothelial activation by inhibiting pro-inflammatory pathways. EMBO Mol. Med..

[37] Raitoharju E., Lyytikäinen L.P., Levula M., Oksala N., Mennander A., Tarkka M., Klopp N., Illig T., Kähönen M., Karhunen P.J. (2011). miR-21, miR-210, miR-34a, and miR-146a/b are up-regulated in human atherosclerotic plaques in the Tampere Vascular Study. Atherosclerosis.

[38] Saba R., Sorensen D.L., Booth S.A. (2014). MicroRNA-146a: A Dominant, Negative Regulator of the Innate Immune Response. Front. Immunol..

[39] Vergadi E., Vaporidi K., Theodorakis E.E., Doxaki C., Lagoudaki E., Ieronymaki E., Alexaki V.I., Helms M., Kondili E., Soennichsen B. (2014). Akt2 deficiency protects from acute lung injury via alternative macrophage activation and miR-146a induction in mice. J. Immunol..

[40] Zhang C., Wang H., Yang B. (2020). miR-146a regulates inflammation and development in patients with abdominal aortic aneurysms by targeting CARD10. Int. Angiol..

[41] Venkatesh P., Phillippi J., Chukkapalli S., Rivera-Kweh M., Velsko I., Gleason T., VanRyzin P., Aalaei-Andabili S.H., Ghanta R.K., Beaver T. (2017). Aneurysm-Specific miR-221 and miR-146a Participates in Human Thoracic and Abdominal Aortic Aneurysms. Int. J. Mol. Sci..

[42] Lareyre F., Clement M., Moratal C., Loyer X., Jean-Baptiste E., Hassen-Khodja R., Chinetti G., Mallat Z., Raffort J. (2019). Differential micro-RNA expression in diabetic patients with abdominal aortic aneurysm. Biochimie.

[43] Hu Y.W., Hu Y.R., Zhao J.Y., Li S.F., Ma X., Wu S.G., Lu J.B., Qiu Y.R., Sha Y.H., Wang Y.C. (2014). An agomir of miR-144-3p accelerates plaque formation through impairing reverse cholesterol transport and promoting pro-inflammatory cytokine production. PLoS ONE.

[44] Araujo N.N.F., Lin-Wang H.T., Germano J.F., Farsky P.S., Feldman A., Rossi F.H., Izukawa N.M., Higuchi M.L., Savioli Neto F., Hirata M.H. (2019). Dysregulation of microRNAs and target genes networks in human abdominal aortic aneurysm tissues. PLoS ONE.

[45] Cerna V., Ostasov P., Pitule P., Molacek J., Treska V., Pesta M. (2019). The Expression Profile of MicroRNAs in Small and Large Abdominal Aortic Aneurysms. Cardiol. Res. Pract..

[46] Kim C.W., Kumar S., Son D.J., Jang I.H., Griendling K.K., Jo H. (2014). Prevention of abdominal aortic aneurysm by anti-microRNA-712 or anti-microRNA-205 in angiotensin II-infused mice. Arterioscler. Thromb. Vasc. Biol..

[47] Holsti M., Wanhainen A., Lundin C., Björck M., Tegler G., Svensson J., Sund M. (2018). Circulating Vascular Basement Membrane Fragments are Associated with the Diameter of the Abdominal Aorta and Their Expression Pattern is Altered in AAA Tissue. Eur. J. Vasc. Endovasc. Surg..

[48] Kuivaniemi H., Ryer E.J., Elmore J.R., Tromp G. (2015). Understanding the pathogenesis of abdominal aortic aneurysms. Expert Rev. Cardiovasc. Ther..

[49] Chen Q., Wang Q., Zhu J., Xiao Q., Zhang L. (2018). Reactive oxygen species: Key regulators in vascular health and diseases. Br. J. Pharmacol..

[50] Ghosh A., DiMusto P.D., Ehrlichman L.K., Sadiq O., McEvoy B., Futchko J.S., Henke P.K., Eliason J.L., Upchurch G.R. (2012). The role of extracellular signal-related kinase during abdominal aortic aneurysm formation. J. Am. Coll. Surg..

[51] Qureshi M.I., Greco M., Vorkas P.A., Holmes E., Davies A.H. (2017). Application of Metabolic Profiling to Abdominal Aortic Aneurysm Research. J. Proteome Res..

[52] Orth M.F., Cazes A., Butt E., Grunewald T.G. (2015). An update on the LIM and SH3 domain protein 1 (LASP1): A versatile structural, signaling, and biomarker protein. Oncotarget.

[53] Choke E., Thompson M.M., Dawson J., Wilson W.R., Sayed S., Loftus I.M., Cockerill G.W. (2006). Abdominal aortic aneurysm rupture is associated with increased medial neovascularization and overexpression of proangiogenic cytokines. Arterioscler. Thromb. Vasc. Biol..

[54] Jones J.A., Zavadzkas J.A., Chang E.I., Sheats N., Koval C., Stroud R.E., Spinale F.G., Ikonomidis J.S. (2010). Cellular phenotype transformation occurs during thoracic aortic aneurysm development. J. Thorac. Cardiovasc. Surg..

[55] Toumpoulis I.K., Oxford J.T., Cowan D.B., Anagnostopoulos C.E., Rokkas C.K., Chamogeorgakis T.P., Angouras D.C., Shemin R.J., Navab M., Ericsson M. (2009). Differential expression of collagen type V and XI alpha-1 in human ascending thoracic aortic aneurysms. Ann. Thorac. Surg..

[56] Chistiakov D.A., Melnichenko A.A., Myasoedova V.A., Grechko A.V., Orekhov A.N. (2017). Thrombospondins: A Role in Cardiovascular Disease. Int. J. Mol. Sci..

[57] Bornstein P., Armstrong L.C., Hankenson K.D., Kyriakides T.R., Yang Z. (2000). Thrombospondin 2, a matricellular protein with diverse functions. Matrix Biol..

[58] Liu J.F., Lee C.W., Tsai M.H., Tang C.H., Chen P.C., Lin L.W., Lin C.Y., Lu C.H., Lin Y.F., Yang S.H. (2018). Thrombospondin 2 promotes tumor metastasis by inducing matrix metalloproteinase-13 production in lung cancer cells. Biochem. Pharmacol..

[59] Helkin A., Maier K.G., Gahtan V. (2015). Thrombospondin-1, -2 and -5 have differential effects on vascular smooth muscle cell physiology. Biochem. Biophys. Res. Commun..

[60] Golledge J., Clancy P., Hankey G.J., Norman P.E. (2013). Relation between serum thrombospondin-2 and cardiovascular mortality in older men screened for abdominal aortic aneurysm. Am. J. Cardiol..

[61] Kato K., Oguri M., Kato N., Hibino T., Yajima K., Yoshida T., Metoki N., Yoshida H., Satoh K., Watanabe S. (2008). Assessment of genetic risk factors for thoracic aortic aneurysm in hypertensive patients. Am. J. Hypertens..

[62] Gellert C., Schottker B., Muller H., Holleczek B., Brenner H. (2013). Impact of smoking and quitting on cardiovascular outcomes and risk advancement periods among older adults. Eur. J. Epidemiol..

[63] Liang B., Che J., Zhao H., Zhang Z., Shi G. (2017). MiR-195 promotes abdominal aortic aneurysm media remodeling by targeting Smad3. Cardiovasc. Ther..

[64] Garcia-Garcia F., Panadero J., Dopazo J., Montaner D. (2016). Integrated gene set analysis for microRNA studies. Bioinformatics.

[65] Mootha V.K., Lindgren C.M., Eriksson K.F., Subramanian A., Sihag S., Lehar J., Puigserver P., Carlsson E., Ridderstråle M., Laurila E. (2003). PGC-1alpha-responsive genes involved in oxidative phosphorylation are coordinately downregulated in human diabetes. Nat. Genet..

[66] Oto J., Navarro S., Larsen A.C., Solmoirago M.J., Plana E., Hervas D., Fernandez-Pardo A., Espana F., Kristensen S.R., Thorlacius-Ussing O. (2020). MicroRNAs and Neutrophil Activation Markers Predict Venous Thrombosis in Pancreatic Ductal Adenocarcinoma and Distal Extrahepatic Cholangiocarcinoma. Int. J. Mol. Sci..

[67] Prado M.S.G., de Goes T.C., de Jesus M.L., Mendonca L.S.O., Nascimento J.S., Kaneto C.M. (2019). Identification of miR-328-3p as an endogenous reference gene for the normalization of miRNA expression data from patients with Diabetic Retinopathy. Sci. Rep..

[68] Breinam L. (2001). Random Forests. Mach. Learn..

[69] Shannon P., Markiel A., Ozier O., Baliga N.S., Wang J.T., Ramage D., Amin N., Schwikowski B., Ideker T. (2003). Cytoscape: A software environment for integrated models of biomolecular interaction networks. Genome Res..

