# Supplementary figures and images for: Identification of Novel microRNA Profiles Dysregulated in Plasma and Tissue of Abdominal Aortic Aneurysm Patients
Source: Int J Mol Sci. 2020 Jun 28;21(13):4600. doi: 10.3390/ijms21134600 (PMC7370113; doi:10.3390/ijms21134600)

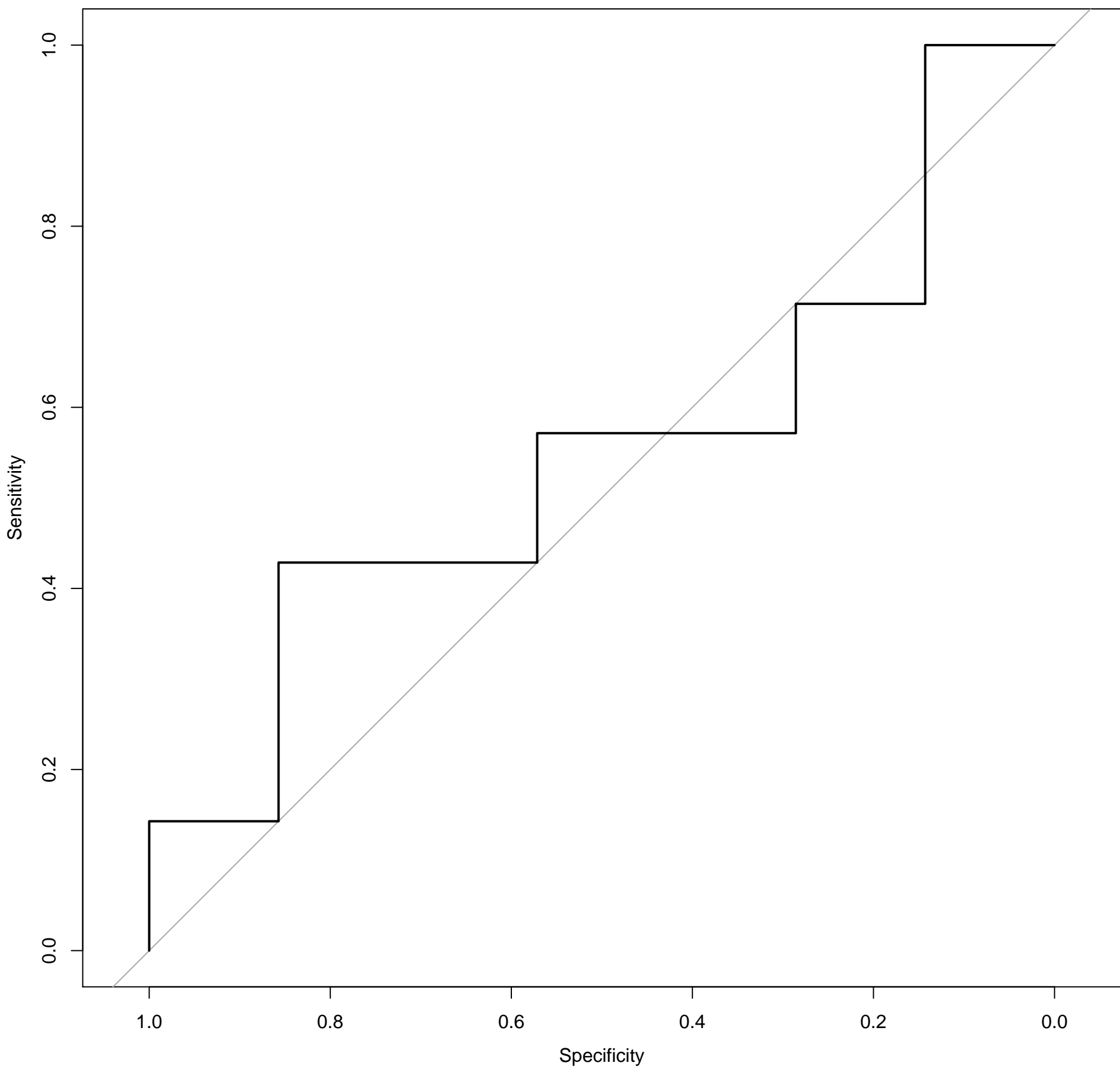

Supplement: Supplementary file 1 [file ijms-21-04600-s001.pdf]
